# Clinical Features and Treatment Strategies of Q Fever Spinal Infection: A Pooled Analysis of 39 Cases and Narrative Review of the Literature

**DOI:** 10.1093/ofid/ofaf584

**Published:** 2025-09-19

**Authors:** Yanhua Liu, Litao Li, Lihui Yang, Yuwei Yang, Huiru An, Dawei Li, Xinjing Wang

**Affiliations:** Senior Department of Tuberculosis, Chinese PLA General Hospital, Beijing, China; Senior Department of Orthopedics, Chinese PLA General Hospital, Beijing, China; Senior Department of Tuberculosis, Chinese PLA General Hospital, Beijing, China; Senior Department of Pulmonary and Critical Care Medicine, Chinese PLA General Hospital, Beijing, China; Senior Department of Tuberculosis, Chinese PLA General Hospital, Beijing, China; Senior Department of Orthopedics, Chinese PLA General Hospital, Beijing, China; Senior Department of Tuberculosis, Chinese PLA General Hospital, Beijing, China

**Keywords:** aneurysm, *coxiella burnetii*, Q fever, spinal infection, tuberculosis

## Abstract

**Background:**

The incidence of spinal infections is increasing; However, pathogen identification remains challenging. Although Q fever spinal infection is reported infrequently, its accrual incidence is likely underestimated. The causative agent, *Coxiella burnetii*, cannot be routinely cultured. Consequently, physicians often misdiagnose Q fever spinal infection as spinal tuberculosis, leading to severe patient harm. Thus, improving clinicians’ awareness of the clinical characteristics of Q fever spinal infection is urgently needed.

**Methods:**

We present a case of Q fever spinal infection and conducted literature searches in PubMed and the Chinese core journals of the Wanfang Database using keywords including “Q fever,” “*Coxiella burnetii*,” “spinal infection,” “osteomyelitis,” “spondylodiscitis,” and “psoas abscess.” Additional reports were identified through cross-referencing, with a cutoff date of 6 November 2024. Cases were included if patient age, sex, and baseline medical history were documented. Clinical data were retrospectively analyzed, and clinical features were compared between the aneurysm-associated group and the isolated spinal infection group. Fisher's exact probability test was used to evaluate the incidence difference.

**Results:**

A total of 39 adult patients were enrolled (mean age: 67.82 ± 10.51 years, male: 34,87.2%), Eleven cases reported potential pathogen exposure. Thirty-three cases presented with early-onset of lower back pain, and 13 developed fever during the disease course. Thirty-four cases involved the lumbar spine, exhibiting continuous lesions of 1–3 vertebral bodies, with imaging features of vertebral osteomyelitis, discitis, paravertebral soft-tissue swelling, and/or adjacent aneurysmal changes. Among 21 cases with routine blood tests, 2 showed elevated leukocyte counts, 5 had mild anemia, and the remainder were normal. Serological testing was performed in 34 cases, with 29 testing positive on the first time; PCR testing was conducted in 25 cases, with 23 cases detecting positive specimens; and rapid diagnosis confirmed in all 3 cases via metagenomic next-generation sequencing (mNGS). Inflammatory reactions were identified in all 21 biopsied cases, with inflammatory granulomas reported in 7 and explicitly excluded in 4. There were 24 cases complicated with aneurysm and 15 cases with isolated spinal infection. A significant difference in CRP elevation rate was observed between the two groups (14/15, 93.33% vs 4/8, 50.00%, *P* = .033). Early local lesion debridement combined with doxycycline-based multidrug therapy showed favorable outcomes. Serological monitoring demonstrated low sensitivity for assessing therapeutic efficacy.

**Conclusions:**

This study systematically summarizes the clinical characteristics of Q fever spinal infection and, for the first time, reports features associated with its distinct clinical subtypes. Q fever should be considered in case of chronic spinal infections—especially those complicated with vascular lesions. Based on clinical history evaluation, rapid diagnosis may be achieved through mNGS of specimens from local lesions. Combined with early initiation of doxycycline-based regimens, timely debridement of necrotic tissues and purulent material may improve treatment outcomes. Further investigations are needed to identify reliable biomarkers for monitoring therapeutic efficacy and to establish optimal treatment strategies for subtypes of Q fever spinal infection.

Spinal infections can affect any part of the spine and paravertebral tissues. Delayed diagnosis and treatment may lead to neurological impairment, paralysis, or death. The incidence of spinal infections has shown a consistent upward trend in recent decades, attributable to multiple factors including increased life expectancy, advancements in diagnostic techniques, more frequent interventional procedures, and the growing prevalence of immunocompromised conditions. Although common spinal infections include pyogenic spondylitis, spinal tuberculosis, and spinal brucellosis, challenges in pathogen identification often result in high rates of misdiagnosis and missed diagnosis [1]. In high-tuberculosis-burden countries, patients presenting with infection-like imaging findings and chronic inflammation on histopathology are frequently treated empirically with anti-tuberculosis therapy. Subsequent disease recurrence and persistence pose substantial socioeconomic burdens.

Recent progress in metagenomic next-generation sequencing (mNGS) technology has markedly improved the diagnostic accuracy for infectious diseases. Using mNGS, we successfully reclassified a treatment-resistant case initially diagnosed as “spinal tuberculosis” to Q fever spinal infection.

Q fever, caused by *Coxiella burnetii* is a globally distributed zoonotic disease. Primary reservoirs include sheep, goats, and cattle, with the pathogen transmission occurring through various secretions such as urine, feces, placental fluids, and milk [2]. Human infections predominantly occur via inhalation of contaminated aerosols, ingestion of infected animal products, or direct mucosal/skin contact with the pathogen [2].

Q fever presents in either acute or chronic infection. While endocarditis was historically considered the exclusive presentation of chronic Q fever, subsequent research has revealed its multi-system involvement, with infective endocarditis accounting for 60%–70% of chronic cases [3]. Contemporary diagnostic and therapeutic approaches for chronic Q fever largely originate from studies focused on Q fever endocarditis [4]. Q fever spinal infection was historically considered uncommon, with only 1 documented case of vertebral osteomyelitis (0.32%) among 313 French chronic Q fever patients diagnosed between 1985 and 1986. However, analysis of the 2016 Dutch National Chronic Q Fever Database demonstrated an 8% incidence rate among 249 patients [5]. This discrepancy likely stems from geographical variations coupled with improvements in diagnostic methodologies and clinical awareness.

Through comprehensive literature analysis, we systematically report Q fever spinal infection and compare clinical features between different subtypes, aiming to establish an evidence-based framework for enhanced detection and optimized treatment strategies. (Figure 1 provides a graphical summary of this study.)

**Figure 1.**
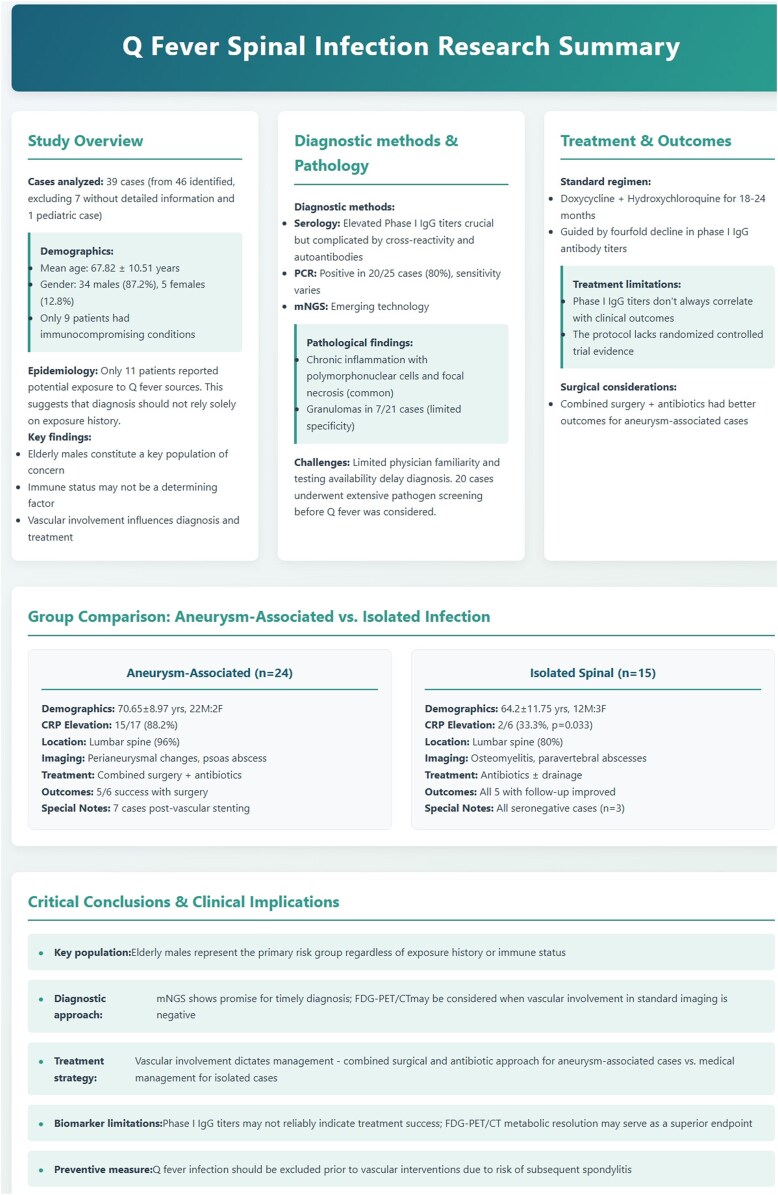
A graphical summary of this study.

## CASE REPORT

A 57-year-old male was admitted on 27 February 2024, presenting with “lower back pain for over 1 year, aggravated with fever for 3 months.” The back pain initially occurred without apparent cause, was relieved by rest, but gradually worsened. Three months prior to admission, he developed an irregular fever up to 38.5°C, accompanied by fatigue and worsening back pain.

At a local hospital, initial tests revealed mild anemia. Computed tomography (CT) scan indicated abdominal aortic aneurysm with a possible retroperitoneal hematoma. He was transferred to Hospital A in Beijing, where vascular surgery was deferred due to fever. Despite 10 days of cefoperazone-sulbactam and vancomycin treatment, his temperature fluctuated between 37.3–37.5°C.

On 2 December 2023, at Hospital B, computed tomography angiography showed an abdominal aortic aneurysm with mural thrombus, surrounded by low-density flocculent shadows, and right psoas muscle enlargement. The lab tests revealed: white blood cell (WBC) count 4.76 × 10^9^/L (neutrophils 55.7%, lymphocytes 27.3%), red blood cell (RBC) count 3.92 × 10^12^/L, hemoglobin 115 g/L, erythrocyte sedimentation rate (ESR) 37 mm/h (0–20), C-reactive protein (CRP) 2.29 mg/ml (0–0.8), fasting glucose 7.7 mmol/L, and procalcitonin 0.04 ng/ml. Brucella agglutination test and multiple blood cultures were negative. Following continued antibiotic therapy, he underwent endovascular aortic repair with bilateral renal artery stenting on December 12. High post-operative fever developed, persisting as low-grade fever after antipyretic treatment.

Over the next ten days, at Hospitals C and D, the interferon-gamma release assay (IGRA) was positive. He was suspected of having lumbar tuberculosis and initiated standard antitubercular-therapy. On 1 February 2024, positron emission tomography/computed tomography (PET/CT) at Hospital E demonstrated increased FDG uptake in L3 vertebra with irregular soft tissue at its right margin. Subsequently, a contrast-enhanced magnetic resonance imaging performed at Hospital F on February 7 revealed post-operative changes from abdominal aortic aneurysm repair at L2-5, along with abnormal signals involving the L2-3 vertebrae and L2-4 paravertebral area. Both imaging suggested possible tuberculosis. However, the patient experienced persistent low back pain and intermittent low-grade fever despite continued anti-tuberculosis therapy. His medical history included diabetes, hypertension and atrophic gastritis. He reported frequently collecting sheep manure from a nearby farm for use as fertilizer in his garden.

On admission, the vitals were stable, and no vascular or cardiac murmurs were heard. The spinal mobility was severely restricted, with tenderness at L2-3. Laboratory tests showed: WBC 5.05 × 10^9^/L (neutrophils 64.9%, lymphocytes 19.4%), RBC 3.8 × 10^12^/L, hemoglobin 104 g/L, CRP 66.31 mg/L (0–10), ESR 77 mm/h (0–15). Fungal markers and blood cultures were negative, IGRA remained positive. CT revealed local bone destruction involving L2–3 vertebrae with right psoas enlargement. Echocardiography showed mild left atrial enlargement and left ventricular diastolic dysfunction, with no valvular vegetations.

On March 1st, CT-guided L3 vertebral biopsy was performed. Fungal and bacterial cultures were negative, but mNGS detected *Coxiella burnetii* (1072 reads, relative abundance 53.55%) with no other pathogens identified. Therefore, anti-tuberculosis therapy was discontinued and treatment with doxycycline (100 mg Bid) + hydroxychloroquine (200 mg Tid) was initiated. on 2 April 2024, CT-guided abscess drainage was performed at Hospital B.

A telephone follow-up on 11 November 2024, indicated that the patient remained compliant with the medication regimen, which was supplemented with traditional Chinese medicine therapy. The patient reported development of a sinus tract at the drainage site with intermittent discharge, along with improved back pain and increased energy levels. Local CT demonstrated stable vertebral lesions without evidence of progression in bony destruction or paravertebral abscess size compared to prior imaging studies.

**Figure 2.**
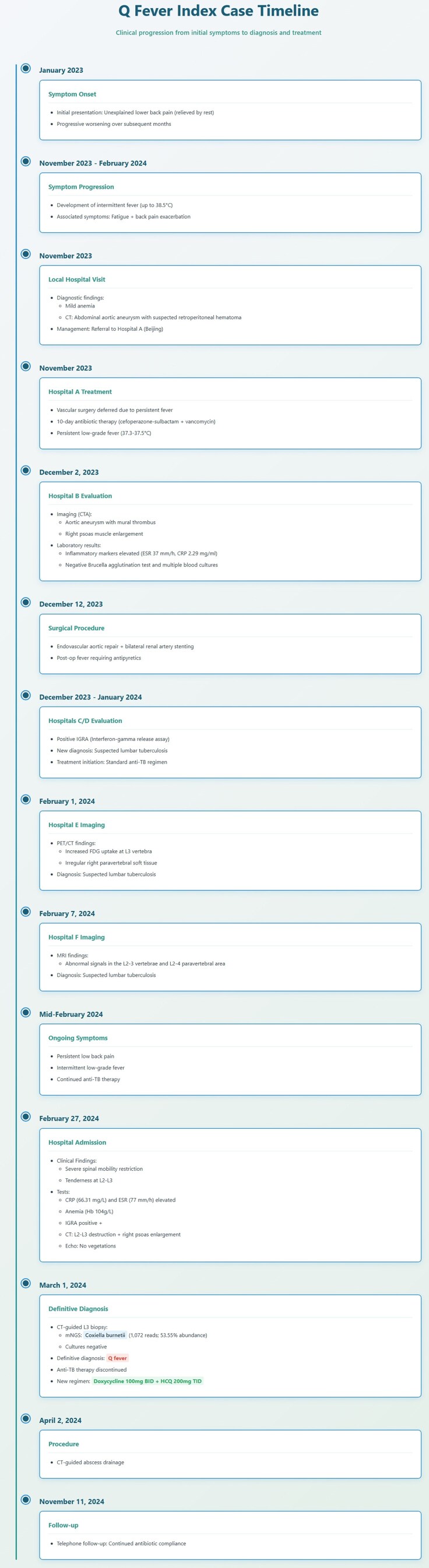
The chronological progression of the index case.

The chronological progression of the index case is detailed in Figure 2.

## RESEARCH METHOD

Building on the comprehensive literature review by Nesrin et al [5] which summarized publications up to March 2020, we expanded the search scope and incorporated recent research advances to conducted a systematic analysis of the clinical characteristics of Q fever spinal infection.

A systematic literature search was performed in PubMed and the Chinese core journals of the Wanfang Database, utilizing keywords such as “Q fever,” “*Coxiella burnetii*,” “spinal infection,” “osteomyelitis,” “spondylodiscitis,” and “psoas abscess.” Additional relevant studies were identified through manual reference screening, with a final search cutoff date of 6 November 2024. Only cases with a confirmed diagnosis of Q fever spinal infection were included, provided they contained documented patient demographics (age, sex) and basic medical history. Clinical characteristics were analyzed, and differences between the two study groups—aneurysm-associated Q fever spinal infection and isolated spinal infection—were assessed. Fisher's exact test was employed to compare the incidence rates of abnormal clinical features between the two groups.

## RESULTS AND DISCUSSION

From 46 identified cases of Q fever spinal infection, 39 cases were included after excluding 7 cases without detailed information and 1 pediatric case [3, 5–29] (demographic, epidemiological, medical history, laboratory, imaging and pathological findings, clinical course, treatment and follow-up outcomes are detailed in Supplementary Tables 1-4). The mean age was 67.82 ± 10.51 years, with 34 males and 5 females. The age and sex distribution aligned with Nesrin's summary [5]. The gender disparity may relate to both occupational exposure and estrogen-enhanced macrophage inflammatory response to *C. burnetii* [30]. The predominance of elderly cases suggests a potential association with age-related immune susceptibility. Only 9 patients reported immunocompromising conditions, including diabetes mellitus (4 cases), chronic kidney disease (2 cases), and malignancies (3 cases). Only 1 patient had suspected acute Q fever history. The relationship between immune status and chronic Q fever remains controversial: some studies suggest immunosuppression and pregnancy as risk factors [9], while others find no significant correlation [3]. Raoult et al [23] reported that all 19 patients who progressed from acute to chronic Q fever had underlying conditions, warranting further investigation into the relationship between immune status and Q fever spinal infection. Epidemiologically, only 11 patients reported potential exposure: 3 raised cattle/sheep, 5 lived in rural areas, and 3 had other animal contacts. Global data indicates Q fever outbreaks frequently occur in urban communities [31], possibly due to diverse transmission routes, high infectivity, and persistent infection characteristics of the pathogen. These findings suggest that Q fever spinal infection diagnosis should not rely overly on exposure history that elderly males constitute a key population of concern, and that immune status may not be a determining factor.

The diagnosis of Q fever traditionally relies on serological testing due to the difficulty in culturing *C. burnetii* and its high infectivity. Elevated Phase I IgG titers are crucial for diagnosing chronic Q fever, however, persistent low titers in specific clinical contexts may also indicate chronic infection [32]. Serological cross-reactivity with other pathogens (eg, *Chlamydia, Legionella, Bartonella, and Brucella*) can complicate interpretation, necessitating additional verification for low-titer positivity. For example, 1 documented case showed weakly positive results for syphilis and brucellosis serology, but these were dismissed due to the absence of clinical symptoms and infection history [20]. Additionally, Q fever may trigger non-specific immune responses, leading to production of autoantibodies (eg, rheumatoid factor, anti-smooth muscle antibodies, or antinuclear antibodies) [33]. One documented case was indeed misdiagnosed as vasculitis due to a prolonged course and positive autoantibodies [11]. Three patients with negative phase I serology were identified. One of these patients was admitted 1 year after the onset of low back pain. Seroconversion occurred after 8 weeks of diagnostic antibiotic therapy, and no molecular testing was performed. The other 2 patients—1 with a documented disease duration of 1 year and the other without detailed duration information—also showed negative serology, but PCR testing of specimens from the local lesion was positive. A notable common feature among these 3 cases was the presence of isolated spinal infection without concomitant vascular involvement. Serological results can be influenced by multiple factors, such as disease stage, host immune status, and pathogen-specific characteristics. However, due to the small number of such cases in our series, it is not possible to draw definitive conclusions regarding the underlying reasons for Sero negativity.

The PCR method for detecting *C. burnetii* DNA in specimens emerged in the 1980s [34]. In 2003, the complete genome sequence of reference strain Nine Mile RSA493.67 became available, enabling more precise molecular diagnostics [34]. The molecular techniques have significantly improved the timeliness of Q fever diagnosis [35], however, sensitivity varies depending on experimental protocols, targeted genomic sequences, and specimen types [32]. Among 25 cases reporting PCR testing, 20 were positive, while 5 showed inconsistent or negative results despite positive serology.

Both PCR and serological methods require clinician awareness. However, limited physician familiarity with Q fever and insufficient availability of standardized testing kits hinder timely diagnosis. In the reviewed cases, 20 cases underwent extensive pathogen screening before Q fever was considered, including tuberculosis testing-often involving repeated specimen collection-and 6 of these patients received prolonged empirical anti-tuberculosis therapy.

In recent years, mNGS technology has played a significant role in pathogen identification due to its untargeted and broad-spectrum characteristics. Three reviewed cases were confirmed through mNGS [11, 22]. Notably, a PubMed search revealed that Chinese scholars have published 35 clinical articles on Q fever, of which 25 were released between 2022 and 2024 and diagnosed via mNGS. However, systematic comparative studies between mNGS and traditional methods remain lacking.

Regarding pathological diagnosis, among 21 cases reporting histopathological findings, chronic inflammation with polymorphonuclear cells and focal necrosis was consistently observed. Seven cases showed inflammatory granulomas, including 1 tuberculosis-like granuloma, while 4 explicitly reported the absence of granulomas. Although some researchers proposed that granulomas, particularly annular granulomas with peripheral fibroblasts, represent characteristic pathological feature of chronic Q fever [3, 9], current data indicate that granulomas are not uniformly present in histopathological examinations, potentially attributable to variations in specimen types and biopsy timing‌.

The standard treatment regimen for chronic Q fever since the 1990s consists of doxycycline-based combination therapy, with hydroxychloroquine serving as the preferred adjunctive agent. Additional therapeutic options include fluoroquinolones, rifamycin, and trimethoprim-sulfamethoxazole. A treatment duration of 18–24 months is recommended, guided by a 4-fold decline in phase I IgG antibody titers. Although this protocol has been adopted in clinical guidelines across multiple countries including France, the Netherlands, and the United States, it primarily derives from retrospective analyses and lacks supporting evidence from randomized controlled trials [4]. In the present study, 3 cases reported treatment durations of 18 months, 2 years, and 3 years respectively, and two cases reported to take lifelong medication. Although a few patients in our series showed clinical improvement without a significant drop in antibody titers, and the large study by Buijs et al [36] similarly found no clear correlation between antibody dynamics and outcomes in Q fever endocarditis, further research is nonetheless needed to define more reliable biomarkers for treatment monitoring. Some researchers suggested FDG PET-CT-confirmed resolution of metabolic activity in lesions may serve as a novel endpoint for treatment [37].

Q fever spinal infection frequently coexists with aortic aneurysm infection. Some researchers propose spinal infection results from vascular infection dissemination [5, 20], while others posit spinal infection extends to involve the aorta [7]. For analytical purposes, we categorized patients into aneurysm-associated and isolated spinal infection groups. Both groups primarily presented with chronic low back pain, with some patients experiencing fever (11/37). Complete blood count results were available for 21 cases, revealing 2 instances of mild leukocytosis, 5 cases of mild anemia, and the remainder with normal blood routine.

The aneurysm-associated group (24 cases) exhibited a marked male predominance (22:2) with a mean age of 70.65 ± 8.97 years. Among 15 cases reporting CRP levels, 14 demonstrated elevation. Lesion distribution showed thoracic spine involvement (T7-9) in 1 case, while the remaining 23 cases presented with lumbar spine involvement, typically affecting 1–2 contiguous vertebrae. The imaging showed perianeurysmal soft tissue changes, psoas abscess, and vertebral erosion.

Regarding treatment, among 6 patients undergoing combined aneurysm repair, abscess debridement, and pharmacotherapy, 1 patient succumbed to surgical complications [19], whereas the remaining 5 achieved favorable recovery during 6–20 months of follow-up. In another 9 cases with pre-existing vertebral erosion and perivascular soft tissue lesions, vascular intervention alone was performed due to initial diagnostic uncertainty, followed by progressive low back pain and vertebral destruction. Unlike tuberculosis, these cases showed no evidence of distant dissemination despite local surgical procedures and prolonged diagnostic delays, suggesting that *C. burnetiid* has a strong local colonization but limited dissemination capacity. This observation underscores the importance of local lesion clearance, as evidenced in the study: among 11 surgically treated cases with complete follow-up, all but 1 achieved positive outcomes, whereas 3 cases antibiotic-only cases developed progressive abscess formation. Of particular clinical significance, 7 patients developed Q fever spondylitis 1–7 years following vascular stenting, emphasizing the necessity of excluding Q fever infection prior to vascular intervention.

The isolated spinal infection group (15 cases) showed a male-to-female ratio of 12:3 and mean age of 64.2 ± 11.75 years. While no cases reported pre-existing immunocompromising conditions, 5 patients had exposure to dairy cattle or lived in rural areas. Radiological findings primarily revealed spinal osteomyelitis and paravertebral abscesses formation, with epidural abscesses observed in 3 cases. Lesion distribution showed lumbar spine involvement in 12 cases (4 extending to S1) and thoracic involvement (T11-12) in 1 case, all affecting 1–3 contiguous vertebrae. In contrast to the consistent CRP elevation in the aneurysm-associated group, only 4 of 8 cases in this group showed elevated CRP levels, demonstrating statistically significant intergroup differences (*P* = .033, Table 1). This finding contrasts with Costa et al's report [25] of universal CRP elevation in 16 Brazilian Q fever cases (without clinical stratification). But due to the limited sample size, this finding should be interpreted with caution.

**Table 1.** Comparison of Clinical Characteristics, Treatment Strategies and Outcomes Between Q Fever Patients With Combined Aortic Aneurysm and Isolated Vertebral Infection

Characteristics	Q Fever With Combined Aneurysm (n = 24)	Q Fever With Isolated Vertebral Infection (n = 15)	*P* Value
Age, mean ± SD, years	70.65 ± 8.97	64.2 ± 11.75	.06
Male, n/N (% [95% CI])	22/24 (91.67 [80.61–102.73])	12/15 (80.00 [59.76–100.24])	.354
Laboratory findings, n/N (% [95% CI])			
Elevated CRP	14/15 (93.33 [80.7–105.96])	4/8 (50.00 [52.28–107.72])	.033^a^
Abnormal blood count	1/10 (10.00 [−8.59–28.59])	1/8 (12.50 [−10.42–35.42])	1
Fever	7/23 (30.43 [11.63–49.23])	6/15 (40.00 [15.21–64.79])	.728
Elevated ESR	6/11 (54.55 [25.12–83.98])	4/8 (50.00 [15.35–84.65])	1
Comorbidities, n/N (% [95% CI])			
Any comorbidity	21/24 (87.50 [74.27–100.73])	14/15 (93.33 [80.7–105.96])	1
Hypertension	11/24 (45.83 [25.9–65.76])	2/15 (13.33 [−3.87–30.53])	.045^a^
Diabetes mellitus	4/24 (16.67 [1.76–31.58])	0/15 (0.00 [0.00–0.00])	.146
Active rheumatoid arthritis	0/24 (0.00 [0.00–0.00])	1/15 (6.67 [−5.96–19.3])	.385
Malignancy	2/24 (8.33 [−2.73–19.39])	1/15 (6.67 [−5.96–19.3])	1
Treatment modality, n/N (% [95% CI])			
Medical therapy alone	3/24 (12.50 [−0.73–25.73])	9/15 (60.00 [35.21–84.79])	.004^a^
Combined medical and surgical	11/24 (45.83 [[25.9–65.76])	4/15 (26.67 [4.29–49.05])	.317
Surgical alone	0/24 (0.00 [0.00–0.00])	0/15 (0.00 [0.00–0.00])	
Follow-up characteristics			
Cases with follow-up, n/N (% [95% CI])	11/24 (45.83 [25.9–65.76])	5/15 (33.33 [9.47–57.19])	.517
Follow-up duration, range, months	6–20	2–36	
Favorable outcome^b^, n/N (% [95% CI])	10/11 (90.91 [73.9–107.9])	5/5 (100.00 [100.00–100.00])	1

Data are presented as n/N, where n is the number of cases with the characteristic and N is the total number of cases with available data for that variable; *P*-values were calculated using Fisher's Exact Test except age, which was analyzed using an independent samples *t*-test.

Abbreviations: CRP, C-reactive protein; ESR, erythrocyte sedimentation rate; 95% CI, 95% confidence interval.

^a^Statistically significant (*P* < .05).

^b^Among patients with follow-up.

Among 13 documented treatment cases, all received continuous antimicrobial therapy, with 4 requiring adjunctive procedures (2 abscess drainages and 2 debridement-decompression surgeries) due to suboptimal pharmacological response. Follow-up data (2–36 months) available for 5 cases showed consistent symptomatic improvement and declining antibody titers.

The pathophysiological relationship between Q fever spinal infection and aortic aneurysm infection remains debated. Our comparative analysis revealed different clinical presentations between groups, suggesting potential differences in infection pathways or pathogen tropism. The presence of vascular infection influences both diagnostic algorithms and therapeutic approaches, making this finding crucial for therapeutic strategy selection. FDG PET/CT exhibits high sensitivity and specificity for vascular infection detection compared to conventional imaging, particularly for identifying occult infectious foci [27]. For Q fever spinal infection cases without vascular abnormalities on standard imaging, PET-CT may help detecting metabolically active lesions indicative of early infection.

Several methodological limitations warrant consideration: (1) selection bias due to exclusion of cases with incomplete demographics, potentially underrepresenting certain subgroups; (2) information bias due to variable data quality across temporal and geographic contexts; (3) publication bias favoring severe/atypical cases; and geographic variability and retrospective design further limit generalizability. Nevertheless, this study maintains scientific value through systematic analysis of the largest reported case series for this rare condition. Diagnostic consistency (all cases confirmed via serology/PCR/mNGS) and transparent acknowledgment of limitations enhance result reliability. While not definitive, it provides foundational insights for clinical practice and future research.

## CONCLUSION

For chronic spinal infections, particularly in elderly males presenting with concurrent aortic aneurysms, Q fever spinal infection should be included in the differential diagnosis. Pathological examination of Q fever spinal infection demonstrates nonspecific features, primarily characterized by chronic inflammation with polymorphonuclear cell infiltration and focal necrosis, occasionally accompanied by granulomatous changes. While serology remains important, molecular methods (PCR and mNGS performed on local specimens) provide critical advantages for rapid pathogen identification, overcoming the limitations of *Coxiella burnetii* phase I IgG antibody detection—particularly in early disease stages or immunocompromised patients. While mNGS is promising in selected settings, its accessibility, cost-effectiveness, and need for clinical correlation should be considered.

The distinct clinical presentations and therapeutic requirements between aneurysm-associated and isolated spinal infection groups necessitate comprehensive vascular evaluation in cases of isolated Q fever spinal infection. Analysis of the aneurysm-cohort revealed that the pathogen *C. burnetii* exhibits a pronounced capacity for ‌local colonization‌ with relatively ‌limited dissemination.‌ These characteristic underscores the ‌critical importance of local lesion debridement‌ for prognosis.

‌This study systematically summarizes the clinical characteristics of Q fever spinal infection and provides, for the first time, a subgroup analysis.‌ Despite the limited sample size, our exploratory findings provide valuable insights into the management of this condition. ‌Future investigations should prioritize‌ the identification of reliable biomarkers for treatment monitoring and the exploration of optimal therapeutic strategies tailored to different subgroups of Q fever spinal infection.

## Supplementary Material

ofaf584_Supplementary_Data

## Supplementary Data

Supplementary materials are available at *Open Forum Infectious Diseases* online. Consisting of data provided by the authors to benefit the reader, the posted materials are not copyedited and are the sole responsibility of the authors, so questions or comments should be addressed to the corresponding author.
