# Supplementary material for: Clinical Features and Treatment Strategies of Q Fever Spinal Infection: A Pooled Analysis of 39 Cases and Narrative Review of the Literature
Source: Open Forum Infect Dis. 2025 Sep 19;12(10):ofaf584. doi: 10.1093/ofid/ofaf584 (PMC12497565; doi:10.1093/ofid/ofaf584)
Supplement: ofaf584_Supplementary_Data [file ofaf584_supplementary_data.zip › Q fever_tableS1.docx]

Supplementary Table S1. Demographic, Epidemiological and Clinical Characteristics of Q Fever Cases

| Case No. | Reference | Coutry | Gender/age | Residence/Animal exposure history | Comorbidity | Symptoms | Associated aneurysm |
| --- | --- | --- | --- | --- | --- | --- | --- |
| 1 | Present case | China | Male/57 | Sheep exposure | HTN,AAA | LBP for 1y, fever for 3m | Yes |
| 2 | [6] | France | Male/70 | Rural | NR | LBP, fever | Yes |
| 3 | [6] | France | Male/70 | Urban | NR | Aneurysm rupture, fever | Yes |
| 4 | [7] | France | Male/64 | NR | S/P aortic graft repair for ruptured AAA | LBP | Yes |
| 5 | [8] | USA | Male/74 | NR | CAD,LSS | LBP | Yes |
| 6 | [9] | USA | Female/60 | Animal exposure 20y prior | HTN,HLP,COPD,AAA,S/p pulmonary carcinoid resection | BP, Weight loss | Yes |
| 7 | [10] | France | Male/65 | NR | CAD,AAA | LBP,Weight loss, fatigue | Yes |
| 8 | [11] | China | Male/79 | NR | HTN,AA | Bilateral subcostal/flank pain | Yes |
| 9 | [12] | China | Male/74 | NR | HTN, DM, HLD | LBP | Yes |
| 10 | [13] | Israel | Male/57 | No animal exposures | NR | LBP | Yes |
| 11 | [5] | Israel | Male/80 | No animal exposures | HTN,COPD,HLP | LBP,fever | Yes |
| 12 | [9] | USA | Male/67 | Farm exposure | AAA | LBP,sweat | Yes |
| 13 | [9] | USA | Male/74 | Farmer | HTN,AAA,CAD | BP | Yes |
| 14 | [14] | Canada | Male/67 | Urban,No animal exposures | HTN, CKD, AA | LBP | Yes |
| 15 | [15] | France | Male/67 | NR | Aneurysm surgery(7y ago) | LBP | Yes |
| 16 | [16] | France | Male/66 | Rural | AAA stent graft (5y ago) | LBP, low-grade fever | Yes |
| 17 | [17] | Northern Ireland | Male/66 | NR | NR | LBP, low-grade fever | Yes |
| 18 | [18] | France | Male/70 | No animal exposures | IAA | LBP, ABP, weight loss | Yes |
| 19 | [5] | Israel | Male/72 | NR | HTN, DM, CAD, PAF | LBP, hematuria×1 | Yes |
| 20 | [19] | Netherlands | Male/77 | NR | AAA | LBP, intermittent claudication | Yes |
| 21 | [20] | France | Male/92 | NR | HTN, DM, CAD | LBP | Yes |
| 22 | [9] | USA | Female/57 | Farm exposure | AAA, mCRC | No | Yes |
| 23 | [5] | Israel | Male/66 | NR | HTN, CAD, CKD, TAA | LBP, weight loss | Yes |
| 24 | [21] | France | Male/91 | NR | HTN, DM, Obesity | LBP, fever | Yes |
| 25 | [22] | China | Male/63 | Farmer | No | LBP,BP, lower extremity weakness | No |
| 26 | [3] | France | Male/47 | NR | History of acute fever 1y ago | R-SN pain | No |
| 27 | [23] | Australia | Female/61 | NR | Bladder Cancer | NR | No |
| 28 | [24] | Scotland | Female/76 | Dairy farmer | No | LBP,fever | No |
| 29 | [24] | Scotland | Male/39 | Dairy farmer | No | LBP,fever | No |
| 30 | [25] | Brazil | Male/67 | NR | NR | LBP,fever | No |
| 31 | [5] | Israel | Female/78 | NR | DLP, OA, CPPD, OP; s/p T12 VP (trauma) 2y ago | Fever,weight loss | No |
| 32 | [26] | France | Male/55 | Near rural | HTN, Obesity | LBP | No |
| 33 | [27] | France | Male/86 | NR | NR | LBP,fatigue | No |
| 34 | [27] | France | Male/66 | NR | NR | LBP, leg pain | No |
| 35 | [27] | France | Male/59 | No animal exposures | NR | LBP | No |
| 36 | [28] | Thailand | Male/64 | Goat dairy farmer | No | LBP | No |
| 37 | [29] | Portugal | Male/72 | Rural,goats exposure | HTN | LBP | No |
| 38 | [3] | France | Male/64 | NR | HCV, OVF, MI, AA, active RA for3y and on TNFi+MTX | LBP,fever | Yes |
| 39 | [10] | France | Male/66 | Intermittent animal exposure | CABG , AA,chronic arteriopathy | LBP,fever | Yes |

**Abbreviation:** AA: Aortic Aneurysm; AAA: Abdominal Aortic Aneurysm; CABG: Coronary Artery Bypass Graft; CAD: Coronary Artery Disease; CKD: Chronic Kidney Disease; COPD: Chronic Obstructive Pulmonary Disease; CPPD: Calcium Pyrophosphate Deposition Disease; DLP: Dyslipidemia; DM: Diabetes Mellitus; HCV: Hepatitis C Virus; HLD: Hyperlipidemia; HLP: Hyperlipidemia; HTN: Hypertension; IAA: Infrarenal Aortic Aneurysm; LBP: Low Back Pain; LSS: Lumbar Spinal Stenosis; mCRC:Metastatic colon adenocarcinoma; MI:Myocardial Infarction; MTX:methotrexate; NR: Not Report; OA: Osteoarthritis; OP: Osteoporosis; OVF: Osteoporotic Vertebral Fracture; RA: Rheumatoid Arthritis; TAA: Thoracic Aortic Aneurysm; TNFi: TNF Inhibitor; PAF:Paroxysmal Atrial Fibrillation
