# Supplementary material for: Clinical Features and Treatment Strategies of Q Fever Spinal Infection: A Pooled Analysis of 39 Cases and Narrative Review of the Literature
Source: Open Forum Infect Dis. 2025 Sep 19;12(10):ofaf584. doi: 10.1093/ofid/ofaf584 (PMC12497565; doi:10.1093/ofid/ofaf584)
Supplement: ofaf584_Supplementary_Data [file ofaf584_supplementary_data.zip › Q fever_tableS2.docx]

Supplementary Table S2. Laboratory, Imaging and Pathological Findings in Q Fever Cases

| Case No. | Reference | WBC  (×109/L) | Hb  (g/dL) | CRP  (normal range) | ESR  (mm/h) | Pathogens screening except Cb | Vertebral involvement | Q fever Phase I Ab | PCR for Cb | mNGS for Cb | PET/CT | Histopathology |
| --- | --- | --- | --- | --- | --- | --- | --- | --- | --- | --- | --- | --- |
| 1 | Present case | 4.7 | NR | 2.29 mg/mL  (<0.8) | 37 | BC(-),mNGS(-),TB-IGRA(+) | L2, L3 | ND | ND | (+) | ND | ND |
| 2 | [6] | 11.7 | NR | NR | 107 | NR | CVO | (+) | ND | NR | NR | NR |
| 3 | [6] | 4.9 | NR | NR | 106 | NR | CVO | (+) | (+) | NR | NR | NR |
| 4 | [7] | NR | NR | NR | NR | MB/fungi/bacteria cx(-) | L3,L4 | (+) | L-spine biopsy (-), iliopsoas abscess (+) | NR | Local and regional infectious process | Vertebral necrosis,Epithelioid granulomatous inflammation |
| 5 | [8] | NR | NR | NR | NR | MB/bacteria/fungi cx(-) |  | (+) | (+) | NR | L5-S1 spondylodiscitis progression with hypermetabolic activity in paraspinous region and right iliac artery aneurysm | NR |
| 6 | [9] | NR | NR | NR | NR | MB/fungi/bacteria cx(-),TB-IGRA(-), fungi/Bartonella sero(-) | L2-4 | (+) | (+) | NR | NR | Chronic inflammation of tissue and bone,Giant cell reaction |
| 7 | [10] | NR | NR | 71 mg/L | 88 | Blood and tissue cx(-), Brucella sero(-) | L4-5 | (+) | (+) | NR | NR | Purulent fluid with PMNs |
| 8 | [11] | Normal | NR | 53.5 mg/L | 25 | TB-IGRA(+) , bacteria/fungi cx(-) | T7-9 | NR | NR | (+) | Irregular soft tissue mass adjacent to thoracic aorta with mild metabolic activity | Diffuse vertebral body necrosis with focal fibrous tissue hyperplasia |
| 9 | [12] | NR | NR | 27.6 g/dL | 32 | AFB/Brucella/fungi cx(-),blood(-),HIV(-),Widal test(-) | L4-5 | (+) | NR | NR | NR | Granulomatous inflammation |
| 10 | [13] | NR | NR | NR | NR | BC(-),HIV(-),Brucella/TP sero(-),AFB/fungi/bacteria cx(-), 16s RNA general PCR(-) | L3-4 | (+) | Bone tissue(+) | NR | NR | Vertebral granuloma with intracellular gram-negative cocci |
| 11 | [5] | NR | 10.5 | 11 mg/dL(<0.5) | NR | BC(+) for S. pneumoniae, tissue cx(+) for K. pneumoniae, P. intermedia, Fusobacterium sp., Brucella sero(-) | L4 , post PA | (+) | Blood and bone tissue(-) | NR | Heterogeneous pathological uptake noted in aortic aneurysmal sac and L4 vertebra | Bone biopsy: Multinucleated inflammatory cell infiltration |
| 12 | [9] | 9.5 | 15.3 | 27.9 mg/dL(<0.8) | 20 | MB/bacteria/fungi cx(-) | L2-3 | (+) | NR | NR | NR | Aortic aneurysm & PA: Caseous necrosis (+) |
| 13 | [9] | 6.4 | 10 | 3.6 mg/dL(<0.8) | 53 | TB/Brucella/Bartonella sero (-),MB/bacteria/fungi cx (-) |  | (+) | aortic and vertebral tissue(+) | NR | NR | Vascular graft: granuloma, neutrophilic inflammation |
| 14 | [14] | NR | 12.5 | 156.8 mg/L | NR | TB/Mycoplasma/Brucella/fungi/bacteria cx(-), HIV/HBV/HCV/TP sero(-) | L1-3 | (+) | Vascular tissue(+) | NR | NR | Normal |
| 15 | [15] | Normal | NR | NR | Normal | BC(-) | L2 | (+) | Vascular and vertebral tissue(+) | NR | NR | NR |
| 16 | [16] | Normal | NR | Normal | Normal | MB/bacteria cx(-) | L2-3 | (+) | NR | NR | NR | NR |
| 17 | [17] | Normall | NR | 20 mg/L | Normal | Bacteria cx(-) | L3-4 | (+) | Vascular graft(+) | NR | NR | NR |
| 18 | [18] | 7 | NR | 34 mg/L | NR | multidrug-resistant Yersinia enterolitica blood cx(+), bacteria tissue cx(-), 16s DNA PCR(-) | L2-3 | (+) | Vascular tissue and abscess(+) | NR | Hypermetabolic foci in endoprosthesis, psoas and L2-L3 spondylodiscitis | Inflammatory infiltration and fibrosis |
| 19 | [5] | NR | NR | Normal | NR | BC(-) | L3 | (+) | ND | NR | Pathological uptake around infrarenal AAA, heterogeneous uptake in L3 vertebra with sclerotic changes and lucent lesions |  |
| 20 | [19] | 5.3 | 14.82 | 12 mg/L(<10） | 22 (<17) | NR | Multiple L-spine |  | blood and CT-guided FNA(+) | NR | FDG-PET(+) | NR |
| 21 | [20] | Normal | DR | 10 mg/L | NR | TB-IGRA(-), TP/Brucella sero(+), bacteria/MB tissue cx(-) | L3 | (+) | ND | NR | NR | Non-caseous necrosis and granulomas, without giant cells |
| 22 | [9] | NR | DR | NR | NR | MB/fungi/bacteria cx(-),TB-IGRA(-), fungi/Bartonella sero(-) | L2-4 | (+) | L-spine and vascular tissue(+) | NR | NR | NR |
| 23 | [5] | Normal | DR | 1.85 mg/dL | NR | BC(-), tissue cx(+）for S. warneri , Brucella sero(-) | L5 , left psoas muscle | (+) | Vascular tissue(+),PA(-) | NR | NR | Chronic inflammation |
| 24 | [21] | Normal | DR | 16 mg/L(<5) | NR | AFB(-) | L3 | (+) | NR | NR | NR | no sign of granulomatosis |
| 25 | [22] | NR | DR | 1.05 mg/L(<10) | 9.9(<15) | BC(-), TB/Brucella sero(-), TST(-) | L1-2 | NR | NR | bone(+) | NR | Inflammatory granuloma |
| 26 | [3] | Normal | DR | NR | NR | BC(-), TST(-),TB/bacteria cx(-) | L5-S1 | (+) | serum and fistula discharge at lumbar puncture site(+) | NR | NR | Inflammatory lesion with tuberculous granuloma |
| 27 | [23] | NR | DR | NR | NR | NR | NR | (+) | NR | NR | NR | NR |
| 28 | [24] | NR | 11.4 | NR | 92 | Brucella(-) | T12-L1 | (+) | NR | NR | NR | NR |
| 29 | [24] | NR | DR | NR | 60 | Brucella(-) | L5 | (+) | NR | NR | NR | NR |
| 30 | [25] | Normal | DR | 50 mg/L | NR | Bartonella/E. chaffeensis sero(-) | NR | (-) → (+) | NR | NR | NR | NR |
| 31 | [5] | NR | 10.2 | 23 mg/dl | NR | Gram stain(-), cx(-) | T11-12 | (+) | Purulent fluid (+) | NR | ND | NR |
| 32 | [26] | NR | NR | <2.9 mg/L | NR | BC(-), tissue cx(-), 16s PCR(-), Brucella/Bartonella sero(-) |  | (+) | Blood and disc cx (-) | NR | L4/5 disc space enhanced uptake | NR |
| 33 | [27] | NR | NR | NR | NR | NR | L2-3 | (-) | Purulent fluid (+) | NR | NR | NR |
| 34 | [27] | NR | NR | NR | NR | NR | L4-S1 | (+) | vertebral tissue(+) | NR | NR | NR |
| 35 | [27] | NR | NR | NR | NR | NR | L5-S1 | (+) | vertebral tissue(+) | NR | NR | NR |
| 36 | [28] | NR | NR | NR | NR | Brucella BC(+), Brucella PCR and MALDI-TOF MS(+), Brucella sero(+) | L3-S1 | (+) | vertebral tissue(+) | NR | NR | Granulomas(-) |
| 37 | [29] | NR | NR | NR | NR | BC(-), Brucella sero(-), TB and HIV(-) | L2-3 | (-) → (+) | NR | NR | NR | NR |
| 38 | [3] | Normal | NR | 33 mg/L | 77 | BC(-), TB cx(-), TST(-) | L2-3 | (+) | Pus/bone(+) | NR | NR | Degenerative lesion, malignancy(-) |
| 39 | [10] | NR | NR | 41 mg/L | 44 | MB/bacteria cx(-),Brucela sero(-), TB PCR(-) | L3-4 | (+) | (+) | NR | NR | Chronic inflammation with PMNs, granuloma (-) |

**Abbreviation:** AAA: Abdominal Aortic Aneurysm; Ab: antibody; sero: serology; AFB: Acid-fast Bacilli; BC: Blood Culture; Cb: Coxiella burnetii; CRP: C-Reactive Protein; CVO: Continuous/Contiguous Vertebral Osteomyelitis; cx: culture; ESR: Erythrocyte Sedimentation Rate; FDG: Fluorodeoxyglucose; FNA: Fine Needle Aspiration; Hb: Hemoglobin; HBV: Hepatitis B Virus; HCV: Hepatitis C Virus; HIV: Human Immunodeficiency Virus; IGRA: Interferon-Gamma Release Assay; MALDI-TOF MS: Matrix-Assisted Laser Desorption/Ionization Time-Of-Flight Mass Spectrometry; MB: Mycobacteria; mNGS:vmetagenomic Next-Generation Sequencing; ND: Not Done; NR: Not Report; PA: Psoas Abscess; PCR: Polymerase Chain Reaction; PET/CT: Positron Emission Tomography/Computed Tomography;PMNs: Polymorphonuclear Cells; TB: Tuberculosis; TP: Treponema Pallidum; TST: Tuberculin Skin Test; WBC: White Blood Cell
