# Supplementary material for: Clinical Features and Treatment Strategies of Q Fever Spinal Infection: A Pooled Analysis of 39 Cases and Narrative Review of the Literature
Source: Open Forum Infect Dis. 2025 Sep 19;12(10):ofaf584. doi: 10.1093/ofid/ofaf584 (PMC12497565; doi:10.1093/ofid/ofaf584)
Supplement: ofaf584_Supplementary_Data [file ofaf584_supplementary_data.zip › Q fever_tableS3.docx]

Supplementary Table S3: Treatment and Follow-up Outcomes of Q Fever Cases

| Case No. | Reference | Empirical treatment before definitive diagnosis | Treatment | Follow-up | Notes |
| --- | --- | --- | --- | --- | --- |
| 1 | Present case | Vanc+ATT | Doxy+HCQ+ AD | 8m treatment, slight improvement in BP |  |
| 2 | [6] | NR | Doxy, 200mg/d | death after 2m |  |
| 3 | [6] | NR | Doxy, 200mg/d | Discontinued medication after 1.5y |  |
| 4 | [7] | NR | Doxy+HCQ | NR |  |
| 5 | [8] | Vanc+PTZ | Doxy + HCQ plus Vertebral vascular surgery |  |  |
| 6 | [9] | Vanc+MEM | Vertebral and vascular surgery,Doxy+Cipro | Phase I IgG antibody titer stable after 4.5y |  |
| 7 | [10] | Antibiotics | Doxy+Oflox+HCQ | Progressive LBP and abscess despite 2m therapy, necessitating surgical debridement |  |
| 8 | [11] | CS+CTX+ATT | Doxy+HCQ+Levo,pain remains unrelieved | death after 6m | APL panel:β2-glycoprotein I IgM: Positive (62.5 AU/mL),Anticardiolipin IgM: Borderline (10.7 MPLU/mL) |
| 9 | [12] | Endovascular stent, psoas abscess aspiration, + CRO 2g + MTZ, + AMC + Levo, + spinal fusion + ATT, LZD (MRSA) | Doxy+HCQ | Good recovery after 2y |  |
| 10 | [13] | Aneurysm surgery + broad-spectrum antibiotics + spinal fusion surgery | Doxy+HCQ | NR |  |
| 11 | [5] | Levo | EVAR followed by persistent lumbar pain, blood cx: S.pneumoniae (+),Levo initiated, persistent low-grade fever, Vertebral biopsy: K. pneumoniae, P. intermedius, Bacteroides sp., suspected AEF, new onset PA,Phase I Ab (+), Moxi+Doxy+HCQ for 2y | 18m post-treatment cessation: Complete serological resolution | Subsequent to PA |
| 12 | [9] | Vanc + FEP + MTZ for 6w，ineffective | Vasc/Vert debrid + (Vanc + Cef + MTZ for 6w)+ Doxy + HCQ | NR |  |
| 13 | [9] | Vanc for 6w | Vanc for 6w, vascular graft removal, soft tissue and lumbar vertebrae debridement, Doxy+HCQ | NR |  |
| 14 | [14] | PTZ + Vanc for 1m | Vascular graft removal, Vanc, PTZ for 1 m, Doxy+HCQ | Titer↓after 20m, died of PE after 22m |  |
| 15 | [15] | NR | Doxy+HCQ for 3w, vascular graft removal and vertebral debridement ,continuous Doxy, HCQ and anticoagulation | Continuous treatment for 3y, good recovery |  |
| 16 | [16] | NR | Simultaneous stent removal, abscess drainage, vertebral fusion, Doxy+HCQ | 6m follow-up, improved |  |
| 17 | [17] | NR | Arterial bypass graft, Doxy + HCQ | Titer↓after 2y | 3y post-operation: Chlamydia Ab(+) and Phase I Ab(+), treated for 3w then discontinued |
| 18 | [18] | NR | Doxy+HCQ for 6m | Good recovery at 6m post-operation |  |
| 19 | [5] | NR | Concurrent endovascular stent with Doxy and HCQ | 18m follow-up, good recovery, Phase I Ab titer unchanged |  |
| 20 | [19] | NR | Surgical reconstruction with spiral saphenous vein graft, Doxy+HCQ | Vascular surgery, intraoperative complications, death |  |
| 21 | [20] | NR | Simultaneous abscess debridement, arterial allograft transplantation, Doxy + HCQ | LBP resolved at 6m |  |
| 22 | [9] | NR | Simultaneous vascular graft, vertebral debridement, Doxy + HCQ | NR |  |
| 23 | [5] | NR | Simultaneous open vascular surgery,Doxy+levo | 6m, good recovery | HGG(21.6%), urine BJP(-) |
| 24 | [21] | NR | Simultaneous vascular debridement,grafting and abscess drainage, Doxy+ HCQ treatment for 18m | Good recovery at 20m |  |
| 25 | [22] | NR | RIF+Doxy+Levo, 1m later, due to worsening pain, underwent aqueous debridement fusion and decompression surgery | NR |  |
| 26 | [3] | ATT | Doxy+HCQ, RIF for 1m | BP↓ and fistula sealed after 1m, vertebral inflammation persists and sero↓ after 1y |  |
| 27 | [23] | NR | NR | NR |  |
| 28 | [24] | ATT | NR | No |  |
| 29 | [24] | NR | TCN treatment for 5m | Symptoms essentially resolved after 5m |  |
| 30 | [25] | Doxy | NR | NR |  |
| 31 | [5] | NR | PSA drainage, Doxy+HCQ | PSA resolved and PD cath out after 8m, good recovery and Ph1 Ab(+) after 1y |  |
| 32 | [26] | NR | Doxy+HCQ | BP↓ and L4-5 uptake resolved after 10m |  |
| 33 | [27] | NR | Doxy+HCQ | NR |  |
| 34 | [27] | NR | Doxy+HCQ | NR |  |
| 35 | [27] | NR | Doxy+HCQ | NR |  |
| 36 | [28] | NR | VDF + Doxy + HCQ + RFP | NR |  |
| 37 | [29] | NR | Doxy + HCQ+AD | NR |  |
| 38 | [3] | ATT | DOX+RIF for 15d, and pain↑, add HCQ+ATT, discontinued after 15d | NR | BP↓, vertebral lesions and abscess reduced improved after 2m |
| 39 | [10] | NR | Doxy+HCQ | Discontinued medication after 3y |  |

**Abbreviation:** Ab: Antibody; AD: Abscess Drainage; AEF: Aortoenteric Fistula; AMC: Metronidazole; APL: Antiphospholipid Antibody; ATT: Anti-TB Therapy; BJP: Bence-Jones Protein; BP: Back Pain; Cb: Coxiella burnetii; Cef: Cefazolin; Cipro: Ciprofloxacin; CRO: Ceftriaxone; CS: Corticosteroids; CTX: Cyclophosphamide; cx: Culture; Doxy: Doxycycline; EVAR: Endovascular Aortic Repair; FEP: Cefepime; HCQ: Hydroxychloroquine; HGG: Hypergammaglobulinemia; LBP: Low Back Pain; Levo: Levofloxacin; LZD: Linezolid; MEM: Meropenem; Moxi: Moxifloxacin; MRSA: Methicillin-Resistant Staphylococcus Aureus; MTZ: Metronidazole; NR: Not Report; Oflox: Ofloxacin; PA: Psoas Abscess; PD: Percutaneous Drainage Catheter; PE: Pulmonary Embolism; PSA: Paraspinal Abscess; PTZ: Piperacillin/Tazobactam; RFP: Rifampicin; RIF: Rifampin; sero: Serology; TB: Tuberculosis; TCN: Tetracycline; Vanc: Vancomycin; Vasc/Vert debrid: Debridement of Vessels and Vertebrae; VDF: Vertebral Debridement and Fusion
