# Supplementary material for: Clinical Features and Treatment Strategies of Q Fever Spinal Infection: A Pooled Analysis of 39 Cases and Narrative Review of the Literature
Source: Open Forum Infect Dis. 2025 Sep 19;12(10):ofaf584. doi: 10.1093/ofid/ofaf584 (PMC12497565; doi:10.1093/ofid/ofaf584)
Supplement: ofaf584_Supplementary_Data [file ofaf584_supplementary_data.zip › Q fever_tableS4.docx]

Supplementary Table S4. Medical history of reported Q fever cases prior to diagnosis

| Case No. | Reference | Clinical presentation and treatment course |
| --- | --- | --- |
| 1 | Present case | Fever and persistent pain after EVAR, unresponsive to anti-tuberculosis therapy |
| 2 | [6] | Post-aneurysm surgery complications |
| 3 | [6] | Post-aneurysm surgery complications |
| 4 | [7] | 9-month history of lumbar pain, worsening after aneurysm surgery (7 months post-operation) |
| 5 | [8] | Pain exacerbation 8 months after aneurysm repair and lumbar laminectomy; empirical treatment with vancomycin and piperacillin for 6 weeks; elevated phase I Q fever antibody titers; vertebral surgery due to progressive pain |
| 6 | [9] | Progressive back pain after aneurysm surgery; underwent spinal and vascular lesion debridement; failed empirical antibiotic therapy |
| 7 | [10] | Persistent abdominal pain one month post-aneurysm surgery; psoas abscess with vertebral erosion; positive phase I antibodies; two months of medical treatment; worsening abdominal pain and abscess progression; no follow-up data available |
| 8 | [11] | Persistent post-aneurysm pain; initially misdiagnosed as systemic vasculitis; treated with methylprednisolone, cyclophosphamide, and analgesics at 7 months post-surgery; lesion progression despite anti-tuberculosis therapy; vertebral biopsy performed at 9 months post-surgery |
| 9 | [12] | Concurrent aneurysm stenting and psoas abscess drainage; treated with ceftriaxone+metronidazole/ertapenem, followed by amoxicillin/clavulanate+levofloxacin; pain recurrence at 6 months; underwent abscess drainage and spinal fusion; subsequent anti-tuberculosis and linezolid therapy; persistent pain leading to repeated spinal fusion |
| 10 | [13] | Open aneurysm surgery after 2 months of broad-spectrum antibiotics; no pathogen identified; progressive lumbar pain; spinal decompression and fusion at 7 months post-aneurysm surgery |
| 11 | [5] | Worsening lumbar pain 2 months post-aneurysm repair; initial blood culture positive for Streptococcus pneumoniae; failed 3-month levofloxacin therapy; repeated vertebral biopsy revealed Klebsiella pneumoniae, Prevotella intermedia, and Bacteroides; progressive psoas abscess |
| 12 | [9] | Intra-abdominal abscess discovered 12 months post-EVAR; abscess progression and vertebral osteomyelitis at 18 months; underwent aneurysm sac and lumbar debridement; empirical treatment with vancomycin, cefepime, and metronidazole for 6 weeks; persistent lumbar pain |
| 13 | [9] | Persistent lumbar pain 2 years post-graft repair; underwent lumbar and epidural abscess debridement; pathogen unidentified until positive phase I Q fever antibodies; subsequent graft removal with lumbar and soft tissue debridement |
| 14 | [14] | Lumbar pain 1 year post-open repair; vertebral inflammation on CT at 4 years; anastomotic pseudoaneurysm diagnosed at 5 years without considering infection; failed 1-month treatment with piperacillin/tazobactam and vancomycin after aneurysm re-repair |
| 15 | [15] | Bilateral hip pain and 4-month history of lumbar pain 7 years post-aneurysm surgery; immediate Q fever testing performed |
| 16 | [16] | Lumbar pain with fever 5 years post-aneurysm surgery; positive phase I Q fever antibodies |
| 17 | [17] | Chlamydia pneumonia and positive phase I Q fever antibodies 3 years post-surgery; treated with doxycycline and ciprofloxacin for 3 weeks; recurrent lumbar pain with fever at 4 years; positive vascular graft C.burnetii PCR |
| 18 | [18] | Lumbar pain 2 years post-surgery; vertebral lesions and psoas abscess; no pathogen identified in vertebral biopsy; positive phase I antibodies |
| 19 | [5] | Incidental abdominal aortic aneurysm; PET-CT showing pathological uptake around aneurysm and L3 vertebral heterogeneous uptake with lucency; elevated phase I Q fever antibodies |
| 20 | [19] | Known abdominal aortic aneurysm with significant enlargement; multiple lumbar vertebral lesions; positive serology and pus PCR |
| 21 | [20] | Concurrent finding of abdominal aortic aneurysm, vertebral and psoas lesions; positive serology |
| 22 | [9] | Diagnosed abdominal aortic aneurysm; progression of intra/extra-aneurysmal and erosive vertebral lesions after 5 months; positive phase I Q fever antibodies |
| 23 | [5] | Positive vascular tissue PCR at time of aneurysm surgery |
| 24 | [21] | Abdominal aortic aneurysm detected during examination; positive phase I antibodies at time of surgery |
| 25 | [22] | Vertebral lesions identified; mNGS testing of biopsy tissue |
| 26 | [3] | Vertebral lesions discovered; biopsy showing "tuberculoid granuloma"; treated with anti-tuberculosis therapy |
| 27 | [23] | No clinical details reported |
| 28 | [24] | Two years of anti-tuberculosis treatment; positive phase I Q fever antibodies overlooked; persistent symptoms |
| 29 | [24] | Brucellosis-like symptoms with single vertebral lesion; negative for Brucella |
| 30 | [25] | Five-day history of fever and spondylitis with vertebral osteomyelitis |
| 31 | [5] | Vertebral lesions and psoas abscess detected; abscess fluid sequencing positive for C. burnetii |
| 32 | [26] | Low back pain; PET/CT showing intense uptake at L4-L5 intervertebral space; positive Q fever serology |
| 33 | [27] | Back pain and fatigue; psoas abscess; culture and PCR positive for C. burnetii from abscess fluid |
| 34 | [27] | Back pain with unilateral leg pain; paravertebral abscess; positive Q fever serology and vertebral biopsy PCR positive for C. burnetii |
| 35 | [27] | Back pain; paravertebral and epidural abscess; positive Q fever serology and vertebral PCR |
| 36 | [28] | Pain symptoms; vertebral decompression surgery; PCR positive for C. burnetii |
| 37 | [29] | Chronic pain; vertebral biopsy; failed empirical treatment |
| 38 | [3] | Vertebral biopsy positive for C. burnetii; symptoms worsened after 15 days of treatment; hydroxychloroquine added; anti-tuberculosis drugs added and discontinued after 15 days; continued doxycycline and hydroxychloroquine |
| 39 | [10] | Positive C. burnetii PCR from abscess fluid; treated |

Abbrevation: C.burnetii: Coxiella burnetii; endovascular aneurysm repair: EVAR; mNGS: Metagenomic Next-Generation Sequencing; PET/CT: Positron Emission Tomography/Computed Tomography; PCR: Polymerase Chain Reaction
