# Supplementary material for: Clinical Features and Treatment Strategies of Q Fever Spinal Infection: A Pooled Analysis of 39 Cases and Narrative Review of the Literature
Source: Open Forum Infect Dis. 2025 Sep 19;12(10):ofaf584. doi: 10.1093/ofid/ofaf584 (PMC12497565; doi:10.1093/ofid/ofaf584)
Supplement: ofaf584_Supplementary_Data [file ofaf584_supplementary_data.zip › renamed_b6271.docx]

Age：

| **Summary Data** | | | | |
| --- | --- | --- | --- | --- |
|  | N | Mean | Std. Deviation | Std. Error Mean |
| Q fever with combined aneurysm | 24.000 | 70.650 | 8.970 | 1.831 |
| Q fever with isolated vertebral infection | 15.000 | 64.200 | 11.750 | 3.034 |

| **Independent Samples Test** | | | | | |
| --- | --- | --- | --- | --- | --- |
|  | Mean Difference | Std. Error Difference | t | df | Sig. (2-tailed) |
| Equal variances assumed | 6.450 | 3.328 | 1.938 | 37.000 | .060 |
| Equal variances not assumed | 6.450 | 3.544 | 1.820 | 24.109 | .081 |
| Hartley test for equal variance: F = 1.716, Sig. = 0.1154 | | | | | |

| **95.0% Confidence Intervals for Difference** | | |
| --- | --- | --- |
|  | Lower Limit | Upper Limit |
| Asymptotic (equal variance) | -.073 | 12.973 |
| Asymptotic (unequal variance) | -.495 | 13.395 |
| Exact (equal variance) | -.294 | 13.194 |
| Exact (unequal variance) | -.862 | 13.762 |

Gender：

| **gender * patients Crosstabulation** | | | | | |
| --- | --- | --- | --- | --- | --- |
|  | | | patients | | Total |
|  |  |  | Q fever with combined aneurysm | Q fever with isolated vertebral infection |  |
| gender | Male | Count | 22 | 12 | 34 |
|  |  | % within gender | 64.7% | 35.3% | 100.0% |
|  | Female | Count | 2 | 3 | 5 |
|  |  | % within gender | 40.0% | 60.0% | 100.0% |
| Total | | Count | 24 | 15 | 39 |
|  |  | % within gender | 61.5% | 38.5% | 100.0% |

| **Chi-Square Tests** | | | | | |
| --- | --- | --- | --- | --- | --- |
|  | Value | df | Asymptotic Significance (2-sided) | Exact Sig. (2-sided) | Exact Sig. (1-sided) |
| Pearson Chi-Square | 1.124^a^ | 1 | .289 |  |  |
| Continuity Correction^b^ | .323 | 1 | .570 |  |  |
| Likelihood Ratio | 1.091 | 1 | .296 |  |  |
| **Fisher's Exact Test** |  |  |  | **.354** | **.280** |
| N of Valid Cases | 39 |  |  |  |  |
| a. 2 cells (50.0%) have expected count less than 5. The minimum expected count is 1.92. | | | | | |
| b. Computed only for a 2x2 table | | | | | |

CRP：

| **CRP * patients Crosstabulation** | | | | | |
| --- | --- | --- | --- | --- | --- |
|  | | | patients | | Total |
|  |  |  | Q fever with combined aneurysm | Q fever with isolated vertebral infection |  |
| CRP | Elevated | Count | 14 | 4 | 18 |
|  |  | % within CRP | 77.8% | 22.2% | 100.0% |
|  | Normal | Count | 1 | 4 | 5 |
|  |  | % within CRP | 20.0% | 80.0% | 100.0% |
| Total | | Count | 15 | 8 | 23 |
|  |  | % within CRP | 65.2% | 34.8% | 100.0% |

| **Chi-Square Tests** | | | | | |
| --- | --- | --- | --- | --- | --- |
|  | Value | df | Asymptotic Significance (2-sided) | Exact Sig. (2-sided) | Exact Sig. (1-sided) |
| Pearson Chi-Square | 5.759^a^ | 1 | .016 |  |  |
| Continuity Correction^b^ | 3.493 | 1 | .062 |  |  |
| Likelihood Ratio | 5.647 | 1 | .017 |  |  |
| **Fisher's Exact Test** |  |  |  | **.033** | **.033** |
| N of Valid Cases | 23 |  |  |  |  |
| a. 2 cells (50.0%) have expected count less than 5. The minimum expected count is 1.74. | | | | | |
| b. Computed only for a 2x2 table | | | | | |

Blood count：

| **Blood_count * patients Crosstabulation** | | | | | |
| --- | --- | --- | --- | --- | --- |
|  | | | patients | | Total |
|  |  |  | Q fever with combined aneurysm | Q fever with isolated vertebral infection |  |
| Blood_count | Abnormal | Count | 1 | 1 | 2 |
|  |  | % within Blood_count | 50.0% | 50.0% | 100.0% |
|  | Normal | Count | 9 | 7 | 16 |
|  |  | % within Blood_count | 56.3% | 43.8% | 100.0% |
| Total | | Count | 10 | 8 | 18 |
|  |  | % within Blood_count | 55.6% | 44.4% | 100.0% |

| **Chi-Square Tests** | | | | | |
| --- | --- | --- | --- | --- | --- |
|  | Value | df | Asymptotic Significance (2-sided) | Exact Sig. (2-sided) | Exact Sig. (1-sided) |
| Pearson Chi-Square | .028^a^ | 1 | .867 |  |  |
| Continuity Correction^b^ | .000 | 1 | 1.000 |  |  |
| Likelihood Ratio | .028 | 1 | .867 |  |  |
| **Fisher's Exact Test** |  |  |  | **1.000** | **.706** |
| N of Valid Cases | 18 |  |  |  |  |
| a. 2 cells (50.0%) have expected count less than 5. The minimum expected count is .89. | | | | | |
| b. Computed only for a 2x2 table | | | | | |

Fever:

| **Fever * patients Crosstabulation** | | | | | |
| --- | --- | --- | --- | --- | --- |
|  | | | patients | | Total |
|  |  |  | Q fever with combined aneurysm | Q fever with isolated vertebral infection |  |
| Fever | Elevated | Count | 7 | 6 | 13 |
|  |  | % within Fever | 53.8% | 46.2% | 100.0% |
|  | Normal | Count | 16 | 9 | 25 |
|  |  | % within Fever | 64.0% | 36.0% | 100.0% |
| Total | | Count | 23 | 15 | 38 |
|  |  | % within Fever | 60.5% | 39.5% | 100.0% |

| **Chi-Square Tests** | | | | | |
| --- | --- | --- | --- | --- | --- |
|  | Value | df | Asymptotic Significance (2-sided) | Exact Sig. (2-sided) | Exact Sig. (1-sided) |
| Pearson Chi-Square | .369^a^ | 1 | .544 |  |  |
| Continuity Correction^b^ | .066 | 1 | .797 |  |  |
| Likelihood Ratio | .367 | 1 | .545 |  |  |
| **Fisher's Exact Test** |  |  |  | **.728** | **.396** |
| N of Valid Cases | 38 |  |  |  |  |
| a. 0 cells (0.0%) have expected count less than 5. The minimum expected count is 5.13. | | | | | |
| b. Computed only for a 2x2 table | | | | | |

ESR：

| **ESR * patients Crosstabulation** | | | | | |
| --- | --- | --- | --- | --- | --- |
|  | | | patients | | Total |
|  |  |  | Q fever with combined aneurysm | Q fever with isolated vertebral infection |  |
| ESR | Elevated | Count | 6 | 4 | 10 |
|  |  | % within ESR | 60.0% | 40.0% | 100.0% |
|  | Normal | Count | 5 | 4 | 9 |
|  |  | % within ESR | 55.6% | 44.4% | 100.0% |
| Total | | Count | 11 | 8 | 19 |
|  |  | % within ESR | 57.9% | 42.1% | 100.0% |

| **Chi-Square Tests** | | | | | |
| --- | --- | --- | --- | --- | --- |
|  | Value | df | Asymptotic Significance (2-sided) | Exact Sig. (2-sided) | Exact Sig. (1-sided) |
| Pearson Chi-Square | .038^a^ | 1 | .845 |  |  |
| Continuity Correction^b^ | .000 | 1 | 1.000 |  |  |
| Likelihood Ratio | .038 | 1 | .845 |  |  |
| **Fisher's Exact Test** |  |  |  | **1.000** | **.605** |
| N of Valid Cases | 19 |  |  |  |  |
| a. 2 cells (50.0%) have expected count less than 5. The minimum expected count is 3.79. | | | | | |
| b. Computed only for a 2x2 table | | | | | |

Comorbidity:

| **Comorbidity * patients Crosstabulation** | | | | | |
| --- | --- | --- | --- | --- | --- |
|  | | | patients | | Total |
|  |  |  | Q fever with combined aneurysm | Q fever with isolated vertebral infection |  |
| Comorbidity | yes | Count | 21 | 14 | 35 |
|  |  | % within Comorbidity | 60.0% | 40.0% | 100.0% |
|  | no | Count | 3 | 1 | 4 |
|  |  | % within Comorbidity | 75.0% | 25.0% | 100.0% |
| Total | | Count | 24 | 15 | 39 |
|  |  | % within Comorbidity | 61.5% | 38.5% | 100.0% |

| **Chi-Square Tests** | | | | | |
| --- | --- | --- | --- | --- | --- |
|  | Value | df | Asymptotic Significance (2-sided) | Exact Sig. (2-sided) | Exact Sig. (1-sided) |
| Pearson Chi-Square | .341^a^ | 1 | .559 |  |  |
| Continuity Correction^b^ | .002 | 1 | .967 |  |  |
| Likelihood Ratio | .360 | 1 | .548 |  |  |
| **Fisher's Exact Test** |  |  |  | **1.000** | **.498** |
| N of Valid Cases | 39 |  |  |  |  |
| a. 2 cells (50.0%) have expected count less than 5. The minimum expected count is 1.54. | | | | | |
| b. Computed only for a 2x2 table | | | | | |

Hypertension:

| **Hypertension * patients Crosstabulation** | | | | | |
| --- | --- | --- | --- | --- | --- |
|  | | | patients | | Total |
|  |  |  | Q fever with combined aneurysm | Q fever with isolated vertebral infection |  |
| Hypertension | yes | Count | 11 | 2 | 13 |
|  |  | % within Hypertension | 84.6% | 15.4% | 100.0% |
|  | no | Count | 13 | 13 | 26 |
|  |  | % within Hypertension | 50.0% | 50.0% | 100.0% |
| Total | | Count | 24 | 15 | 39 |
|  |  | % within Hypertension | 61.5% | 38.5% | 100.0% |

| **Chi-Square Tests** | | | | | |
| --- | --- | --- | --- | --- | --- |
|  | Value | df | Asymptotic Significance (2-sided) | Exact Sig. (2-sided) | Exact Sig. (1-sided) |
| Pearson Chi-Square | 4.387^a^ | 1 | .036 |  |  |
| Continuity Correction^b^ | 3.047 | 1 | .081 |  |  |
| Likelihood Ratio | 4.764 | 1 | .029 |  |  |
| **Fisher's Exact Test** |  |  |  | **.045** | **.038** |
| N of Valid Cases | 39 |  |  |  |  |
| a. 0 cells (0.0%) have expected count less than 5. The minimum expected count is 5.00. | | | | | |
| b. Computed only for a 2x2 table | | | | | |

Diabetes mellitus:

| **Diabetes_mellitus * patients Crosstabulation** | | | | | |
| --- | --- | --- | --- | --- | --- |
|  | | | patients | | Total |
|  |  |  | Q fever with combined aneurysm | Q fever with isolated vertebral infection |  |
| Diabetes_mellitus | yes | Count | 4 | 0 | 4 |
|  |  | % within Diabetes_mellitus | 100.0% | 0.0% | 100.0% |
|  | no | Count | 20 | 15 | 35 |
|  |  | % within Diabetes_mellitus | 57.1% | 42.9% | 100.0% |
| Total | | Count | 24 | 15 | 39 |
|  |  | % within Diabetes_mellitus | 61.5% | 38.5% | 100.0% |

| **Chi-Square Tests** | | | | | |
| --- | --- | --- | --- | --- | --- |
|  | Value | df | Asymptotic Significance (2-sided) | Exact Sig. (2-sided) | Exact Sig. (1-sided) |
| Pearson Chi-Square | 2.786^a^ | 1 | .095 |  |  |
| Continuity Correction^b^ | 1.269 | 1 | .260 |  |  |
| Likelihood Ratio | 4.166 | 1 | .041 |  |  |
| Fisher's Exact Test |  |  |  | .146 | .129 |
| N of Valid Cases | 39 |  |  |  |  |
| a. 2 cells (50.0%) have expected count less than 5. The minimum expected count is 1.54. | | | | | |
| b. Computed only for a 2x2 table | | | | | |

Active rheumatoid arthritis

| **Active_rheumatoid_arthritis * patients Crosstabulation** | | | | | |
| --- | --- | --- | --- | --- | --- |
|  | | | patients | | Total |
|  |  |  | Q fever with combined aneurysm | Q fever with isolated vertebral infection |  |
| Active_rheumatoid_arthritis | yes | Count | 0 | 1 | 1 |
|  |  | % within Active_rheumatoid_arthritis | 0.0% | 100.0% | 100.0% |
|  | no | Count | 24 | 14 | 38 |
|  |  | % within Active_rheumatoid_arthritis | 63.2% | 36.8% | 100.0% |
| Total | | Count | 24 | 15 | 39 |
|  |  | % within Active_rheumatoid_arthritis | 61.5% | 38.5% | 100.0% |

| **Chi-Square Tests** | | | | | |
| --- | --- | --- | --- | --- | --- |
|  | Value | df | Asymptotic Significance (2-sided) | Exact Sig. (2-sided) | Exact Sig. (1-sided) |
| Pearson Chi-Square | 1.642^a^ | 1 | .200 |  |  |
| Continuity Correction^b^ | .058 | 1 | .810 |  |  |
| Likelihood Ratio | 1.953 | 1 | .162 |  |  |
| **Fisher's Exact Test** |  |  |  | **.385** | **.385** |
| N of Valid Cases | 39 |  |  |  |  |
| a. 2 cells (50.0%) have expected count less than 5. The minimum expected count is .38. | | | | | |
| b. Computed only for a 2x2 table | | | | | |

Malignancy

| **Malignancy * patients Crosstabulation** | | | | | |
| --- | --- | --- | --- | --- | --- |
|  | | | patients | | Total |
|  |  |  | Q fever with combined aneurysm | Q fever with isolated vertebral infection |  |
| Malignancy | yes | Count | 2 | 1 | 3 |
|  |  | % within Malignancy | 66.7% | 33.3% | 100.0% |
|  | no | Count | 22 | 14 | 36 |
|  |  | % within Malignancy | 61.1% | 38.9% | 100.0% |
| Total | | Count | 24 | 15 | 39 |
|  |  | % within Malignancy | 61.5% | 38.5% | 100.0% |

| **Chi-Square Tests** | | | | | |
| --- | --- | --- | --- | --- | --- |
|  | Value | df | Asymptotic Significance (2-sided) | Exact Sig. (2-sided) | Exact Sig. (1-sided) |
| Pearson Chi-Square | .036^a^ | 1 | .849 |  |  |
| Continuity Correction^b^ | .000 | 1 | 1.000 |  |  |
| Likelihood Ratio | .037 | 1 | .848 |  |  |
| **Fisher's Exact Test** |  |  |  | **1.000** | **.674** |
| N of Valid Cases | 39 |  |  |  |  |
| a. 2 cells (50.0%) have expected count less than 5. The minimum expected count is 1.15. | | | | | |
| b. Computed only for a 2x2 table | | | | | |

Medical therapy alone

| **Medical_therapy_alone * patients Crosstabulation** | | | | | |
| --- | --- | --- | --- | --- | --- |
|  | | | patients | | Total |
|  |  |  | Q fever with combined aneurysm | Q fever with isolated vertebral infection |  |
| Medical_therapy_alone | yes | Count | 3 | 9 | 12 |
|  |  | % within Medical_therapy_alone | 25.0% | 75.0% | 100.0% |
|  | no | Count | 21 | 6 | 27 |
|  |  | % within Medical_therapy_alone | 77.8% | 22.2% | 100.0% |
| Total | | Count | 24 | 15 | 39 |
|  |  | % within Medical_therapy_alone | 61.5% | 38.5% | 100.0% |

| **Chi-Square Tests** | | | | | |
| --- | --- | --- | --- | --- | --- |
|  | Value | df | Asymptotic Significance (2-sided) | Exact Sig. (2-sided) | Exact Sig. (1-sided) |
| Pearson Chi-Square | 9.777^a^ | 1 | .002 |  |  |
| Continuity Correction^b^ | 7.674 | 1 | .006 |  |  |
| Likelihood Ratio | 9.870 | 1 | .002 |  |  |
| **Fisher's Exact Test** |  |  |  | **.004** | **.003** |
| N of Valid Cases | 39 |  |  |  |  |
| a. 1 cells (25.0%) have expected count less than 5. The minimum expected count is 4.62. | | | | | |
| b. Computed only for a 2x2 table | | | | | |

Combined medical and surgical:

| **Combined_medical_surgical * patients Crosstabulation** | | | | | |
| --- | --- | --- | --- | --- | --- |
|  | | | patients | | Total |
|  |  |  | Q fever with combined aneurysm | Q fever with isolated vertebral infection |  |
| Combined_medical_surgical | yes | Count | 11 | 4 | 15 |
|  |  | % within Combined_medical_surgical | 73.3% | 26.7% | 100.0% |
|  | no | Count | 13 | 11 | 24 |
|  |  | % within Combined_medical_surgical | 54.2% | 45.8% | 100.0% |
| Total | | Count | 24 | 15 | 39 |
|  |  | % within Combined_medical_surgical | 61.5% | 38.5% | 100.0% |

| **Chi-Square Tests** | | | | | |
| --- | --- | --- | --- | --- | --- |
|  | Value | df | Asymptotic Significance (2-sided) | Exact Sig. (2-sided) | Exact Sig. (1-sided) |
| Pearson Chi-Square | 1.433^a^ | 1 | .231 |  |  |
| Continuity Correction^b^ | .737 | 1 | .391 |  |  |
| Likelihood Ratio | 1.468 | 1 | .226 |  |  |
| **Fisher's Exact Test** |  |  |  | **.317** | **.196** |
| N of Valid Cases | 39 |  |  |  |  |
| a. 0 cells (0.0%) have expected count less than 5. The minimum expected count is 5.77. | | | | | |
| b. Computed only for a 2x2 table | | | | | |

Surgical alone:

| **Surgical_alone * patients Crosstabulation** | | | | | |
| --- | --- | --- | --- | --- | --- |
|  | | | patients | | Total |
|  |  |  | Q fever with combined aneurysm | Q fever with isolated vertebral infection |  |
| Surgical_alone | no | Count | 24 | 15 | 39 |
|  |  | % within Surgical_alone | 61.5% | 38.5% | 100.0% |
| Total | | Count | 24 | 15 | 39 |
|  |  | % within Surgical_alone | 61.5% | 38.5% | 100.0% |

| **Chi-Square Tests** | |
| --- | --- |
|  | Value |
| Pearson Chi-Square | .^a^ |
| N of Valid Cases | 39 |
| a. No statistics are computed because Surgical_alone is a constant. | |

Cases with follow-up:

| **Cases_with_follow_up * patients Crosstabulation** | | | | | |
| --- | --- | --- | --- | --- | --- |
|  | | | patients | | Total |
|  |  |  | Q fever with combined aneurysm | Q fever with isolated vertebral infection |  |
| Cases_with_follow_up | yes | Count | 11 | 5 | 16 |
|  |  | % within Cases_with_follow_up | 68.8% | 31.3% | 100.0% |
|  | no | Count | 13 | 10 | 23 |
|  |  | % within Cases_with_follow_up | 56.5% | 43.5% | 100.0% |
| Total | | Count | 24 | 15 | 39 |
|  |  | % within Cases_with_follow_up | 61.5% | 38.5% | 100.0% |

| **Chi-Square Tests** | | | | | |
| --- | --- | --- | --- | --- | --- |
|  | Value | df | Asymptotic Significance (2-sided) | Exact Sig. (2-sided) | Exact Sig. (1-sided) |
| Pearson Chi-Square | .596^a^ | 1 | .440 |  |  |
| Continuity Correction^b^ | .191 | 1 | .662 |  |  |
| Likelihood Ratio | .603 | 1 | .438 |  |  |
| **Fisher's Exact Test** |  |  |  | **.517** | **.333** |
| N of Valid Cases | 39 |  |  |  |  |
| a. 0 cells (0.0%) have expected count less than 5. The minimum expected count is 6.15. | | | | | |
| b. Computed only for a 2x2 table | | | | | |

Lost to follow-up:

| **Lost_to_follow_up * patients Crosstabulation** | | | | | |
| --- | --- | --- | --- | --- | --- |
|  | | | patients | | Total |
|  |  |  | Q fever with combined aneurysm | Q fever with isolated vertebral infection |  |
| Lost_to_follow_up | yes | Count | 13 | 10 | 23 |
|  |  | % within Lost_to_follow_up | 56.5% | 43.5% | 100.0% |
|  | no | Count | 11 | 5 | 16 |
|  |  | % within Lost_to_follow_up | 68.8% | 31.3% | 100.0% |
| Total | | Count | 24 | 15 | 39 |
|  |  | % within Lost_to_follow_up | 61.5% | 38.5% | 100.0% |

| **Chi-Square Tests** | | | | | |
| --- | --- | --- | --- | --- | --- |
|  | Value | df | Asymptotic Significance (2-sided) | Exact Sig. (2-sided) | Exact Sig. (1-sided) |
| Pearson Chi-Square | .596^a^ | 1 | .440 |  |  |
| Continuity Correction^b^ | .191 | 1 | .662 |  |  |
| Likelihood Ratio | .603 | 1 | .438 |  |  |
| Fisher's Exact Test |  |  |  | .517 | .333 |
| N of Valid Cases | 39 |  |  |  |  |
| a. 0 cells (0.0%) have expected count less than 5. The minimum expected count is 6.15. | | | | | |
| b. Computed only for a 2x2 table | | | | | |

Favorable outcome:

| **Favorable_outcome * patients Crosstabulation** | | | | | |
| --- | --- | --- | --- | --- | --- |
|  | | | patients | | Total |
|  |  |  | Q fever with combined aneurysm | Q fever with isolated vertebral infection |  |
| Favorable_outcome | yes | Count | 10 | 5 | 15 |
|  |  | % within Favorable_outcome | 66.7% | 33.3% | 100.0% |
|  | no | Count | 1 | 0 | 1 |
|  |  | % within Favorable_outcome | 100.0% | 0.0% | 100.0% |
| Total | | Count | 11 | 5 | 16 |
|  |  | % within Favorable_outcome | 68.8% | 31.3% | 100.0% |

| **Chi-Square Tests** | | | | | |
| --- | --- | --- | --- | --- | --- |
|  | Value | df | Asymptotic Significance (2-sided) | Exact Sig. (2-sided) | Exact Sig. (1-sided) |
| Pearson Chi-Square | .485^a^ | 1 | .486 |  |  |
| Continuity Correction^b^ | .000 | 1 | 1.000 |  |  |
| Likelihood Ratio | .779 | 1 | .377 |  |  |
| **Fisher's Exact Test** |  |  |  | **1.000** | **.688** |
| N of Valid Cases | 16 |  |  |  |  |
| a. 3 cells (75.0%) have expected count less than 5. The minimum expected count is .31. | | | | | |
| b. Computed only for a 2x2 table | | | | | |

Poor_outcome:

| **Poor_outcome * patients Crosstabulation** | | | | | |
| --- | --- | --- | --- | --- | --- |
|  | | | patients | | Total |
|  |  |  | Q fever with combined aneurysm | Q fever with isolated vertebral infection |  |
| Poor_outcome | yes | Count | 1 | 0 | 1 |
|  |  | % within Poor_outcome | 100.0% | 0.0% | 100.0% |
|  | no | Count | 10 | 5 | 15 |
|  |  | % within Poor_outcome | 66.7% | 33.3% | 100.0% |
| Total | | Count | 11 | 5 | 16 |
|  |  | % within Poor_outcome | 68.8% | 31.3% | 100.0% |

| **Chi-Square Tests** | | | | | |
| --- | --- | --- | --- | --- | --- |
|  | Value | df | Asymptotic Significance (2-sided) | Exact Sig. (2-sided) | Exact Sig. (1-sided) |
| Pearson Chi-Square | .485^a^ | 1 | .486 |  |  |
| Continuity Correction^b^ | .000 | 1 | 1.000 |  |  |
| Likelihood Ratio | .779 | 1 | .377 |  |  |
| **Fisher's Exact Test** |  |  |  | **1.000** | **.688** |
| N of Valid Cases | 16 |  |  |  |  |
| a. 3 cells (75.0%) have expected count less than 5. The minimum expected count is .31. | | | | | |
| b. Computed only for a 2x2 table | | | | | |
